# The Dermatologic Care Needs of a Rural Community in South Florida

**DOI:** 10.3390/ijerph20043071

**Published:** 2023-02-09

**Authors:** Sara M. Asbeck, Brenda U. Imo, Okelue E. Okobi, Jennifer Dorcé-Medard

**Affiliations:** 1 Dr. Phillip Frost Department of Dermatology and Cutaneous Surgery, University of Miami, Miami, FL 33136, USA; 2 Division of Dermatology, Georgetown University School of Medicine, Washington, DC 20007, USA; 3 Lakeside Medical Center, Belle Glade, FL 33430, USA

**Keywords:** dermatology, public health, rural health, remote health, minorities, healthcare access, environmental health, occupational health, skin of color, skin cancer

## Abstract

For patients in rural areas, primary care is often their only access to healthcare services, and skin concerns are among the most common diseases seen in these settings. This study aims to investigate the most common skin conditions, management trends and patterns of referral to dermatology in a rural and underserved community in South Florida. A retrospective chart review was conducted using medical records from the C.L. Brumback Primary Care Clinic in Belle Glade, FL. The most common skin conditions were fungal infections, unspecified dermatitis, pruritus, skin cancer concern, alopecia, and autoimmune skin disorders. The most frequent management strategy was medication prescription followed by specialist referral. Of the 21 percent of patients referred to a specialist, 55 percent of these were to dermatology. The most common diagnoses referred to dermatology were atopic dermatitis and alopecia. Only 20 percent of these patients reported attending their follow-up appointment, and the average distance to referral was 21 miles. Belle Glade is unique in its need for and access to dermatologic care. The lack of access to specialists in rural communities is a public health issue that more studies and outreach initiatives should address.

## 1. Introduction

Rural populations have substantial issues with regards to healthcare access, especially that of specialized healthcare providers. The city of Palm Beach has one of highest incomes per capita among all Florida towns, and yet 40 miles west lies Belle Glade, a town with some of the worst unemployment and poverty in the country [1]. In 2018, 42.1 percent of the Belle Glade population was living below the poverty line, over three times the national average of 13.1 percent [2]. The predominant ethnic groups in the area are Black or African American (58 percent), Hispanic (30.4 percent), and Non-Hispanic White (7.67 percent), and the largest industries are the agriculture, forestry, fishing, and hunting industries [2].

The rural nature of Belle Glade presents unique challenges to the provision of dermatologic care in this region. For patients in rural areas, primary care is often their only access to healthcare services, and skin concerns are among the most common diseases seen in these settings [3]. Studies have shown, however, that compared to primary care physicians (PCPs), dermatologists are much more accurate when diagnosing skin conditions, and ‘far superior’ at detecting melanoma [3]. In rural areas, PCPs often refer patients to specialists that are far away or rely on unofficial recommendations from specialist colleagues and friends. Studies on access to care demonstrate that even for free healthcare, patients would likely not travel more than 20 miles [3], and for a population where over a third of people are living below the poverty line, driving to seek care is likely a huge barrier to access.

Another unique feature in this community is that nearly 90 percent of the Belle Glade population is composed of racial and ethnic minorities. According to the National Ambulatory Medical Care Survey, the top diagnoses for African American dermatology patients are acne, unspecified dermatitis or eczema, seborrheic dermatitis, atopic dermatitis, dyschromia, psoriasis, alopecia, and keloid scars [4]. While acne and unspecified dermatitis or eczema are common diagnoses in all racial and ethnic groups in the U.S., dyschromia and certain types of alopecia are much more commonly encountered in patients with skin of color. This group is also distinct in that treatment delays and misdiagnoses can be extremely detrimental, leading to sequelae such as hypertrophic scarring, keloid formation, post-inflammatory hyperpigmentation, and permanent hair loss [4]. The minority population is also historically less likely to seek specialist care. For the sole diagnosis of a skin condition, 43.2 percent of Caucasians said they would visit a dermatologist, while only 28.5 percent of African Americans reported amenability [5]. 

This study aims to investigate the need for dermatologic care in the medically underserved and majority African American community of Belle Glade, FL. A retrospective chart review was performed to investigate the rates and patterns of skin diseases in this region as well as the frequency and pattern of dermatology referrals in this patient population. Ultimately, we hope to use the information from this study to characterize the scarcity of dermatologic care in this area in order to inform ways in which we might improve access in this and other resource-limited communities.

## 2. Materials and Methods

A retrospective single-center study was conducted at the Lakeside Medical Center, which houses the Family Medicine Residency Clinic (C.L. Brumback Primary Care Clinics) in Belle Glade, FL. The study protocol was reviewed by the Nova Southeastern University Institutional Review Board and, based on the nature of the study, granted exemption from requiring ethics approval and written informed consent. Patient medical records from 1 January 2020 to 2 January 2022 meeting the following criteria were included: (1) at least one clinic visit coded with ICD-10 diagnosis of one of the conditions listed in the AAD Dermatology ICD-10-CM Quick Coder Guide; (2) 18 years or older at time of the initial visit. Patient records were reviewed by a study team member. The first round of review identified the initial clinic visit of interest. Records for patients under 18 years old, duplicate records, and records for conditions that are not typically managed by a dermatologist (such as vaginal candidiasis) were excluded. In the second round, qualitative and quantitative data were extracted from the clinic visit note including patient age, sex, race, ethnicity, diagnosis, number of related follow-ups, actions taken by physician, medications prescribed, referrals given, and the outcome of the referral if noted. Any uncertainty in chart inclusion or data collection was resolved through discussion between study team members. Data analysis was performed with the SPSS 28.0 statistical package. Descriptive statistics were calculated for all categories.

## 3. Results

### 3.1. Patient Demographics

Of the 935 patients included in this study, there was a female predominance (61 percent) compared to males (39 percent). The mean patient age was 48 years (ranging from 10 to 92 years). Considering patients’ racial distribution, the majority identified as Black or African-American (50 percent) and Hispanic or Latino (43 percent). Less than 1 percent of patients did not report their racial identification. All patient characteristics are listed in Table 1.

### 3.2. Diagnosis and Management

The most common patient diagnoses were fungal infection (35 percent), unspecified dermatitis (24 percent), pruritus (13 percent), skin cancer concern (5 percent), alopecia (4 percent), and autoimmune skin disorder (4 percent). The main actions taken by the treating PCP were prescribing medication (73 percent) and referring to a specialist (21 percent). Of the 895 medications prescribed, the majority were anti-fungals (53 percent), corticosteroids (21 percent), and antibacterials (14 percent). The average number of related clinic follow-ups was 2. Patient clinical characteristics are listed in Table 2.

### 3.3. Outcome

**Figure 1:**
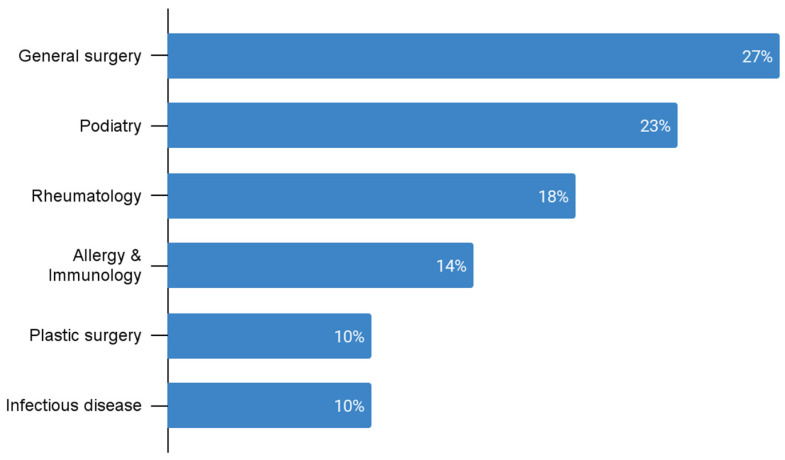
Most referred specialties other than dermatology (n = 22).

**Figure 2:**
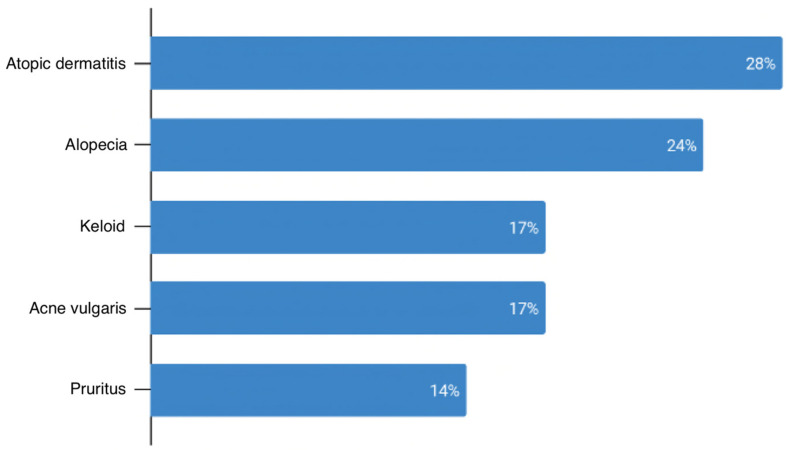
Most common diagnoses referred to dermatology (n = 29).

**Figure 3:**
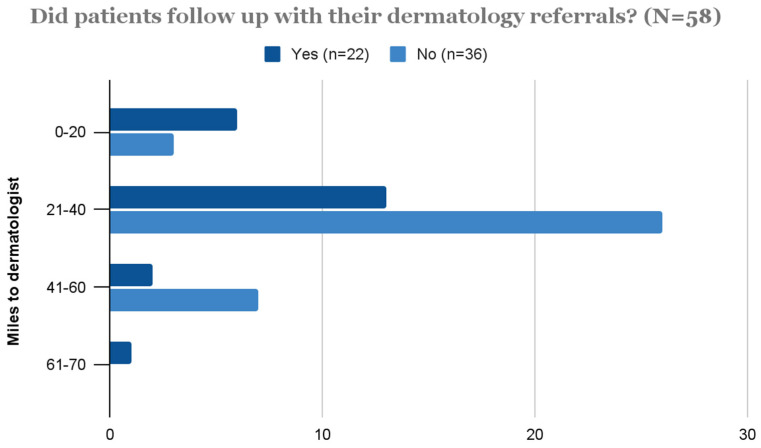
Patients’ follow-up trends and distance to referred dermatologists (Fisher–Freeman–Halton Exact Test, *p* = 0.092).

One hundred and thirty-three patients received specialist referrals; 55 percent of these were to dermatology and 46 percent to other specialties. The most common non-dermatology referrals were for general surgery (6), podiatry (5), and rheumatology (4) (Figure 1). The most common diagnoses referred to dermatology were atopic dermatitis (8) and alopecia (7) (Figure 2). Of the 109 patients that received dermatology referrals, 20 percent reported attending their follow-up appointment while 40 percent did not attend their follow-up appointment. Follow-up attendance was not reported for approximately 40 percent of patients. The average distance to patients’ referred dermatologists was 21 miles. The distance to referred dermatologists was not indicated in the chart for 10 patients. The majority of patients that did not follow up with their dermatology referrals lived approximately 21–30 miles from the clinic (Figure 3). A Fisher–Freeman–Halton Exact test found no difference between patients in the “Yes” and “No” follow-up categories and distance to referred dermatologists, *p* = 0.092.

## 4. Discussion

Belle Glade is a predominantly rural agricultural community, and our findings show that the most common dermatologic conditions affecting the patients of this region were fungal infections, unspecified dermatitis, and pruritus. The hot and humid conditions farm workers face in South Florida, combined with prolonged and repeated exposure to pests, pesticides, and other irritants provide the perfect environment for skin irritation, inflammation and infection [6]. Similar to Belle Glade, rural America is predominantly farmland populated by agricultural laborers, and employees are continuously exposed to extreme humidity during farm chores. Multiple researchers have demonstrated the link between excessive humidity and fungal diseases [6,7,8,9,10,11,12,13,14] The most common pesticide-related skin disease is contact dermatitis, and less common diseases include urticaria, erythema multiforme, ashy dermatosis, occupational acne, porphyria cutanea tarda, hair and nail disorders, and skin cancer [6]. Guo et al. [7], in an occupational and environmental study to demonstrate the prevalence of pesticide-associated dermatoses in Southern Taiwan, an area similar in humidity to Belle Glade, observed a high prevalence of pesticide-related skin disease [6]. Similarly, the CDC and several other authors have documented similar pesticide-related skin diseases amongst farmers [10,11,12,13,14].

An issue somewhat unique to the Belle Glade area and other regions of the country where sugar cane burning is prevalent is increased air pollution. For six to eight months a year, smoke and ash engulf the town as cane farmers burn their harvest [7]. This is likely a contributing factor to the higher rates of skin irritation and atopic dermatitis in this region, as air pollutants have been implicated in the pathogenesis of eczema and respiratory allergic diseases [14]. It is important to note that because patients under the age of 18 were excluded from our study, the rates of atopic dermatitis are grossly underestimated in this sample but it was a common diagnosis in the excluded pediatric charts.

Alopecia was within the top 10 dermatologic conditions experienced by our population. Alopecia is a common diagnosis in the skin of color population, partially due to genetics, although environmental conditions and hair care practices are thought to have an influence on its prevalence. Inflammatory processes as well as physical and emotional stress lead to a misbalance between reactive oxygen species and antioxidants, leading to oxidative tissue damage [14]. For alopecia areata, oxidative damage is thought to induce the disruption of the immune privilege status of the hair follicle and promote autoimmunity [14]. In central centrifugal cicatricial alopecia, a genetic mutation has been identified which increases hair follicle susceptibility to inflammation and eventual destruction [15]. In addition, a substantial number of patients presented with skin cancer concerns. Agricultural workers are at increased risk for skin cancer, primarily due to excess sunlight exposure as well as pesticides [6,10,11,12,13,14,15,16,17]. This remains an urgent and ongoing concern for primary care clinics that see a large population of farmers and migrant workers. Early detection and prevention will be key to decreasing the burden of disease and associated costs of skin cancer in this population.

In terms of management, the discovery that 73 percent of patient visits were managed by the PCP prescribing a medication implies that for those diagnoses often seen in the primary care clinic such as infection or dermatitis, most PCPs felt comfortable managing the patient themselves. Interestingly, of the 21 percent of patient visits that were referred to a specialist, nearly half of the referrals went to non-dermatology providers. Given the scarcity of dermatology providers in this region, other specialists such as general surgeons, podiatrists, and rheumatologists have likely become adept in managing several dermatological conditions that have cross-over into their specialties. The top conditions that were referred to dermatology were atopic dermatitis, alopecia, keloids, and acne. Severe cases of these conditions can be complex, have a huge impact on patients’ quality of life, and may be beyond the scope of practice for a PCP.

Only 20 percent of patient charts denoted that referrals were completed. This is unsurprising, given the average referral distance of 21 miles, and that almost half of the patients in this region live below the poverty line and do not have access to reliable transportation. Distance to referral was not significantly correlated to referral follow-through, although this may have been affected by the nearly 40 percent of patient charts that did not indicate referral completion.

Cited barriers to health care services among the general and vulnerable populations include transportation, insurance status, legal status, financial costs, language barriers, cultural barriers, intimidation by healthcare settings, and hours of operation [6,7,8,9,10,11,12,13,14,15,16,17]. Options for navigating these barriers and bridging the access gap to specialists in these rural communities include telemedicine, visiting consultants, and mobile clinics [17]. Telemedicine, in particular, is a particularly efficient way for PCPs to access specialty services with neither patient nor provider needing to travel. Studies have shown that less than 7% of malignant skin lesions are missed by teledermatology [18]. If a specialist is willing to travel, visiting consultations have the potential to improve health outcomes and reduce unnecessary hospital-based interventions, especially when combined with education of local primary care providers [19].

One major limitation of the study was that the COVID-19 pandemic occurred during the years studied. This likely influenced patient referral and follow-up trends, as non-emergency patient visits declined dramatically at the height of the pandemic. Another limitation was that the Lakeside Medical Center changed its electronic medical record system in 2020, so some of the documentation in the older charts was found to be incomplete and subsequently excluded from the study, decreasing the total sample size.

## 5. Conclusions and Recommendations

During the study period, there was a high prevalence of dermatologic presentations, with the majority being fungal and dermatitis-related. Access to dermatologist specialist services is difficult for patients in rural areas to obtain, and there are high levels of non-compliance with referrals due to the distance from the nearest dermatologist specialist. Several reports have documented problems with access to care in rural communities, which are also often left out of traditional research. Our study brings awareness to this population and similar populations across the country. Although other rural populations in America may resemble our study population in terms of resources, availability of specialist providers, and environmental conditions, what makes Belle Glade especially unique is the large population of minorities and immigrants living in the area, which likely impacts the conditions they experience as well as residents’ degree of engagement with the healthcare system. The lack of access to resources in rural communities is a huge public health issue and one that more studies and outreach programs need to address. More research is required to understand the factors underlying these gaps in dermatologic services in rural communities. In future studies of this population, we intend to collect qualitative data via questionnaires and focus group discussions to inquire about physician comfort level in treating specific conditions, patient willingness/ability/desire to see a dermatologist, and acceptance and feasibility of some of the methods for delivering dermatologic care to this rural community.

We recommend that dermatologists investigate alternative methods of providing dermatologic care, such as forming strong partnerships with rural primary care providers, developing an efficient referral system, maximizing the potentials of telemedicine, and employing mobile clinics to make their practice as accessible to these vulnerable populations as possible.

## Figures and Tables

**Table 1:** Patient characteristics (n = 935).

Variable	n (%) or M ± SD
Sex	
Female	568 (61)
Male	367 (39)
Age	48 ± 14
Race	
American Indian or Alaska Native	1 (0.1)
Asian	5 (0.5)
Black or African-American	463 (50)
Hispanic or Latino	400 (43)
Native Hawaiian or Other Pacific Islander	1 (0.1)
White	64 (7)
Unreported	1 (0.1)

Percentages have been rounded and may not total 100.

**Table 2:** Clinical characteristics.

Variable	n (%) or M ± SD
Top 10 patient diagnoses (n = 726)	
Fungal infection	257 (35)
Dermatitis, unspecified	173 (24)
Pruritus	93 (13)
Benign skin growth	55 (8)
Skin cancer concern	36 (5)
Alopecia	29 (4)
Autoimmune skin disorder	29 (4)
Acne vulgaris	21 (3)
Skin and soft tissue infection	17 (2)
Scabies	16 (2)
Top 5 Actions taken by Physician (n = 935)	
Medication prescribed	680 (73)
Referral provided	200 (21)
Labs Ordered	54 (6)
OTC product recommended	34 (4)
In-office procedure performed *	32 (3)
Top 5 Medications prescribed (n = 831)	
Anti-fungal	441 (53)
Corticosteroid	175 (21)
Anti-bacterial	116 (14)
Antihistamine	68 (8)
Anti-inflammatory	31 (4)

Percentages have been rounded and may not total 100. * Skin biopsy, cryotherapy, incision and drainage, aspiration, excision.

## Data Availability

The data presented in this study are available on request from the corresponding author.
